# Trustworthy Wireless Sensor Networks for Monitoring Humidity and Moisture Environments

**DOI:** 10.3390/s21113636

**Published:** 2021-05-24

**Authors:** Radomir Prodanović, Sohail Sarang, Dejan Rančić, Ivan Vulić, Goran M. Stojanović, Stevan Stankovski, Gordana Ostojić, Igor Baranovski, Dušan Maksović

**Affiliations:** 1 Centre for Applied Mathematics and Electronics, Serbian Armed Forces, 11000 Belgrade, Serbia; radomir.prodanovic@vs.rs; 2 Faculty of Technical Sciences, University of Novi Sad, 21000 Novi Sad, Serbia; sohail@uns.ac.rs (S.S.); sgoran@uns.ac.rs (G.M.S.); stevan@uns.ac.rs (S.S.); goca@uns.ac.rs (G.O.); 3 Faculty of Electronic Engineering, University of Niš, 18000 Niš, Serbia; dejan.rancic@elfak.ni.ac.rs (D.R.); dusan.maksovic@skysoft.rs (D.M.); 4 Military Academy, University of Defence, 11000 Belgrade, Serbia; ivan.vulic@mod.gov.rs

**Keywords:** trust, sensor networks, humidity and moisture sensors, monitoring application, timestamp, public key infrastructure (PKI)

## Abstract

Wireless sensors networks (WSNs) are characterized by flexibility and scalability in any environment. These networks are increasingly used in agricultural and industrial environments and have a dual role in data collection from sensors and transmission to a monitoring system, as well as enabling the management of the monitored environment. Environment management depends on trust in the data collected from the surrounding environment, including the time of data creation. This paper proposes a trust model for monitoring humidity and moisture in agricultural and industrial environments. The proposed model uses a digital signature and public key infrastructure (PKI) to establish trust in the data source, i.e., the trust in the sensor. Trust in data generation is essential for real-time environmental monitoring and subsequent analyzes, thus timestamp technology is implemented here to further ensure that gathered data are not created or changed after the assigned time. Model validation is performed using the Castalia network simulator by testing energy consumption at the receiver and sender nodes and the delay incurred by creating or validating a trust token. In addition, validation is also performed using the Ascertia TSA Crusher application for the time consumed to obtain a timestamp from the free TSA. The results show that by applying different digital signs and timestamps, the trust entity of the WSN improved significantly with an increase in power consumption of the sender node by up to 9.3% and receiver node by up to 126.3% for a higher number of nodes, along with a packet delay of up to 15.6% and an average total time consumed up to 1.186 s to obtain the timestamp from the best chosen TSA, which was as expected.

## 1. Introduction

The development of the Internet of Things (IoT) has significantly increased the demand for wireless sensor networks (WSNs) in both industry and academia. WSNs allow sensors from multiple environments to be connected for different purposes. The flexibility and scalability of WSNs allows application in various fields, such as agriculture, health [1,2], industry, transport, farming, and the military [3,4]. WSNs can generally be applied in any environment where there is a need for data collection and a need to manage systems in the environment.

Intensive agricultural and industrial practices require the application of modern technology to monitor and manage environments. The use of sensors significantly contributes to the creation of the most optimal environment for plants and animals in agricultural production. The optimal environment for growth and cultivation is created by managing environmental parameters such as temperature, humidity, soil moisture, brightness, CO_2_, and solar radiation [5,6]. Sensors are also used in the feeding process, monitoring diseases, and pests in agriculture.

Sensors automatically collect environmental data and send them to monitoring stations over a wired or wireless network in industrial production contexts. Production processes that require expensive production operations and raw materials that are monitored by sensors require a reliable and confidential data transmission network. Monitoring and controlling such processes requires a network with mechanisms that enable reliability and a high quality of communication [7,8], as well as the availability of data, authenticity, and confidentiality [9]. Aponte-Luis et al. designed an efficient WSN for monitoring and control in an industrial environment [10]. The system met industry requirements and was designed so that it could be extended to measure other parameters. The design of the monitoring system included sensors, communication between the sensor and the base station, transmission from the base station to the monitoring system, and data storage. The system supported energy budget management and required minimal maintenance.

It is necessary to establish a system consisting of sensors that will collect data, gateways that will transmit data, and monitoring applications [11,12,13] to develop an appropriate system for monitoring humidity and moisture in agricultural and industrial contexts. Data may be stored in a database and analyzed. A decision regarding the treatment of the observed environment is made based on the obtained results from the analysis, i.e., starting actions that make changes in the environment (an increase of humidity, humidity, or other monitored parameters). After recollecting the data, a decision may be made regarding further action for the observed environment [14,15].

We need to trust sensors to be sure that we obtain accurate data for humidity or moisture. It is not enough to gain trust just by looking at the physical existence of sensors in the observed environment, but we have to know that the data come from real sensors. The design and implementation of a monitoring system has not been discussed in earlier papers [5,10,11,12,13,14,15,16,17,18,19], which have not considered establishing mutual trust between the sensor, base station, and monitoring application.

The trust of WSN sensors in IoT contexts may be considered from the aspect of recognizing a reliable sensor, communication, and data by calculating metrics to determine the degree of trust in the sensor [20,21,22,23,24,25,26]. Another way to achieve trust is by applying security through general measures, PKI, and cryptography (blockchain) [27,28,29]. The time dimension has not been taken into account in previous papers related to trust in WSNs and environment monitoring. For a confidential, reliable, and accurate analysis of environmental conditions, it is necessary that the information for the time of data generation is unchanged. It is important to have trust in the source and time of data creation, especially if the analysis or prediction is carried out based on previously collected data. Reliable analysis and efficient decision-making should be based on trust that the data originate from the right sensor, that they have not been changed, and that there is evidence of occurrence at the recorded time.

The authors of this paper consider sensor trust and monitoring applications at the time of data exchange. The paper proposes a model for trustworthy data exchange between entities in a WSN for moisture and humidity monitoring using asymmetric cryptography, PKI, and timestamping. The model was developed based on the IoT trust model [30]. It is based on a trust check considering the submitted sensor data on the receiver side. It is possible to trust data that were not created or changed after the assigned time by applying timestamping in the model. The model does not depend on the parameters observed in the environment, but can also be applied to sensors that collect other data, such as brightness, CO_2_, solar radiation, and other parameters in an industrial environment or any other observed environment.

The main contributions of this work are the introduction of the time dimension of trust in a humidity and moisture sensor monitoring environment, as well as the extended trust that provides certainty in the reliability of data for subsequent analyses. The data are not changed and data are not created after the assigned time. The rest of the paper is organized as follows. The next section presents papers in the field of monitoring agricultural and industrial environments and models of trust in the context of the IoT. Section 3 describes the applied materials and methods. Section 4 describes a model of a trusted wireless sensor network for monitoring humidity and moisture. Section 5 provides the simulation results and evaluation of the proposed model using the Castalia simulator and Ascertia TSA Crusher application. Section 6 provides the conclusions of this work. 

## 2. Related Work

### 2.1. Agriculture and Industry Monitoring Environment

Gaikwad et al. [5] designed and developed a WSN to monitor conditions in an agricultural environment. The system monitored various parameters, such as nitrates, zinc, potassium, phosphorus, humidity, and temperature. The research was conducted using a WiFi network for remote monitoring with WSN802G modules for collecting data from sensors. The sensor data were stored on a central server and then analyzed and displayed. The system can be used in agriculture, greenhouse management, industrial environment control, and industrial fertilizer contexts.

In [11], the authors proposed a new hardware design for sensor nodes and a software execution flow diagram for the needs of an agriculture environment monitoring system. Experiments have shown that the system is energy-efficient, stable, and highly accurate. The system can be used for real-time monitoring of an agricultural environment.

The design, development, and implementation of a WSN based on the Zigbee protocol was proposed in [12]. The WSN collects data from sensors and delivers it to a central node that forwards the data to a central monitoring station using the General Packet Radio Service (GPRS) or Global System for Mobile Communications (GSM) standards. The system also sends sensor position data to a central monitoring station.

Muangprathub et al. [13] developed a system for elucidating the optimal irrigation of crops in agriculture using sensors controlled via smartphones and a web application. The system consisted of hardware, a web application, and a mobile application. The basic hardware component is a soil moisture sensor used to collect data in the field. The web application component analyzes the data collected by the sensors and determines the optimal conditions for crop growth, such as the temperature, humidity, and soil moisture. The user manages crop irrigation using the mobile application.

Smart farming, precision agriculture, or smart agriculture are new agricultural techniques that enable the intensive production of agricultural products. These agricultural techniques require the application of modern communication and information technology to ensure product quality. Autonomous plant growing systems use soil moisture sensors to increase irrigation system energy and time efficiency and reduce water consumption. The IL-69 soil moisture sensor has been used to automate a sprinkler system and has been applied in [14]. Sensor values may be displayed on an LCD screen and on a website in real time.

A model for crop monitoring using soil moisture sensors and humidity levels was proposed in [15]. The information is transmitted via a Zigbee network to a remote computer for monitoring conditions. The remote computer manages devices to ensure an optimal environment for crops based on the received information and the particular decision.

Humidity sensors play a crucial role in WSNs applied to precision agriculture. The requirements for humidity sensors and a comparative review of research papers in the field of the design and modeling of humidity sensor performance have been given in [16]. Characteristically, the requests do not require sensor trust or the time when the data are collected.

Rahul et al. [17] presented the results of a WSN assessment in a real industrial environment. They showed the application of two industrial WSNs for the real-time collection of data in industrial applications. They used an Atmega32u4 acquisition system to collect data. This system generated a large amount of data to be sent to the monitoring destination. The authors applied a cloud solution using an open-source API.

The authors in [18] gave an example of a sensor application for measuring humidity and temperature. They designed and implemented a WSN network to monitor relative humidity and temperature using sensors, microcontrollers, and Xbee modules. The data were sent to a host computer which served as a monitoring station. Nooriman et al. [19] presented a WSN for monitoring environmental parameters in a Harumanis mango orchard using temperature, humidity, and soil moisture sensors. The system used a star topology to connect the sensor to a base station connected to a data storage server. Both papers dealt with technical solutions without the consideration of trust.

### 2.2. Trust Models

The authors in [20] considered different trust models that monitor sensor network behavior and calculate metrics that can be used to assess network attacks. They described the main design issues and discussed existing trust models used in various wireless sensor network decision-making processes.

The authors in [21] proposed a trust assessment model and trust-based data fusion mechanism. This model consisted of three types of trust: behavioral trust, data trust, and historical trust. Total trust is obtained by a weighted calculation which generates a list of trust scores. The trust list guarantees the reliability of the data included in the fusion process. In their work, the list was initialized to contain all sensor nodes and changes over time according to the calculated confidence of the sensor node.

Jiang et al. [22] proposed an efficient distributed trust model (EDTM) for WSNs. Direct trust and recommended trust were calculated based on the number of packets received by the node sensor. Direct trust was calculated based on communication trust, energy trust, and data trust. Trust reliability and familiarity were used to improve the accuracy of the recommended trust. This model more accurately assessed the reliability of sensor nodes and more effectively prevented security breaches.

An unreliable node in a network can cause significant damage and negatively affect the quality and reliability of data. Determining the level of trust for a node has a positive effect on the trust of an entity to perform a transaction through the node. The authors in [23] presented different approaches for computing trust in mobile ad hoc networks (MANETs). They also analyzed approaches to trust propagation dynamics, prediction, and aggregation algorithms, along with the impact of network dynamics on trust dynamics and the impact of trust on security services.

Direct trust is calculated based on sensor node communication in most trust models. Such models do not take the harmful consequences of link quality on trust into account. To reduce the harmful consequences on the connectivity of the nodes, Wu et al. [24] proposed the introduction of a beta and link quality indicator (LQI)-based trust model (BLTM) for WSNs. The LQI analysis mechanism made it possible to maintain the trust values of normal nodes and provide accuracy in a real network environment. Direct trust was calculated based on trust in communication, trust in energy, and trust in data. They then discussed the stated trusts in order to calculate the most accurate direct trust and thus the reliable trust value for the sensor node.

An attacker can physically manipulate sensor nodes by entering incorrect or false data through them to disrupt the normal operation of nodes. Chen et al. [25] have developed a distributed agent-based trust model in WSNs to solve the problem of malicious nodes. The model used a watchdog scheme to observe the behavior of nodes and broadcast their trust ratings.

Rathore et al. [26] presented a new approach to calculate sensor nodes trust using sociopsychological norms, benevolence, and integrity. They proposed two models: a model that can be used in a WSN to calculate the reliability of sensor nodes and a model for removing nodes with a reliability below a certain threshold. Errors that occur after information is sent reduce sensor node trust. Transmission errors also reduce the trust for a given sensor node. A low-trust node is considered less authentic and credible, so it should be removed.

Lu et al. applied PKI and cryptography in an authentication protocol for vehicular ad hoc networks (VANETs) [27]. The authors have developed a system that prevents vehicles from broadcasting counterfeit messages while maintaining privacy. The authors proposed a blockchain-based anonymous reputation system (BARS) to establish a trust model for VANETs. Certificates and a certificate revocation process were applied effectively as proof of vehicle presence or absence using extended blockchain technology. The public keys were used as pseudonyms in communication to hide the real identity. The results showed that BARS established trust with transparency, conditional anonymity, efficiency, and robustness for VANETs.

She et al. [28] presented a trust model that detects malicious WSN nodes. The model is based on cryptographic blockchain technology. They described the whole framework for the model and then built a blockchain data structure that detects malicious nodes. Finally, they realized the detection of malicious nodes in 3D space by using a blockchain smart contract and the WSN quadrilateral measurement localization method.

The authors of [29] proposed an IoT architecture that includes a general systematic network and application security through basic requests of data safety. They did not elaborate on how the IoT integrates with digital signature technology and timestamps.

A detailed overview of different trust models for WSNs and an analysis of different applications of trust models are given in [31]. The authors considered trust models in ordinary WSNs and trust models in cluster-based WSNs. The comparison of trust models was performed through the following parameters: methodology, trust values, advantages, performance limitations, and complexity.

Prodanović et al. [32] proposed a data security model that could be used in various agricultural monitoring applications. The model is based on the application of asymmetric and symmetric cryptography for data security and PKI. The model includes practical aspects, changes in the sensor node architecture, and optimization of the model by applying organizational and technical measures. These aspects were considered in order to preserve data security while saving energy.

The application of trustworthiness by a public key and digital signature has been considered in [33]. The trust was transmitted through transitivity via nodes, i.e., by the creation of a trust chain. IoT entities from the same or different environments may have certificates issued by different certification authorities. It was not considered how to establish trust between entities with certificates issued by different certification authorities. Also, the time factor of trustworthiness was not considered. 

## 3. Materials and Methods

The objective of this research is to create a WSN trust model for moisture and humidity monitoring in agricultural and industrial environments. We have studied various solutions for monitoring WSNs in agricultural and industrial environments and models of WSN trust in IoT contexts to achieve this objective. For different trust models in WSNs, we examined applications of a WSN in agricultural and industrial environments and researched and applied symmetric and asymmetric cryptography, PKI, and timestamps for trust model development. 

For the literature research, the following databases were used: ABI/INFORM Global, Academic Search Premier, ACM Digital Library, Applied Science & Technology Full Text (EBSCO), IEEE Xplore, ScienceDirect, and Google Scholar.

We applied a digital signature and digital certificate to build trust between entities. The digital signature used asymmetric cryptography, i.e., a private key to generate the digital signature and a public key to verify it. The digital certificate binds the public key to the entity identity and represents the electronic identification of the entity. A public key is associated with a corresponding private key. The PKI generates and manages digital certificates such that any other entity in the environment can validate the connection between the certificate and the identity [34]. The digital certificates used for identity management were based on X.509 certificates, which describe entities based on the ANS.1 specification. X.509 certificates are encoded using the DER standard and stored as an ASCII string using BASE64 encoding [35].

A hash function compresses input content into output content that is significantly smaller in size. A cryptographic hash function has special characteristics that make it suitable for cryptographic use. Some of these functions belong to the family of one-way functions. Most cryptographic hash functions are designed to take a string of any length as an input and produce a fixed-length hash value which is random and cannot be converted back into the input.

A digital signature is a mathematical scheme for the realization of authentication, non-repudiation, and message integrity. It is necessary to have pairs of keys (private and public cryptographic keys) to realize digital signatures. Digital signature schemes typically consist of the follow two algorithms [30]:

- An algorithm for signing. This algorithm generates a digital signature for a given message and a private key;
- An algorithm for verification of the digital signature. This algorithm verifies or denies whether a message is authentic based on the message, public key, and signature.

The stamping service consists of a set of principals with a timestamping authority (TSA) and a publishing authorization, along with quadruple (S, C, V, P) protocols. A participant uses protocol S to send a TSA timestamp request and protocol C receives a timestamp from the TSA. Verification algorithm V is used to confirm the authenticity of the timestamp and the time event sequences. Publishing protocol P uses the TSA to publish the timestamps on an authenticated and easily accessible medium [36].

A WSN for monitoring of agriculture conditions was designed with the Flora framework of Omnet++ software. We evaluated the performance of the proposed model in terms of energy consumption at the receiver and sender nodes and packet delay using the Castalia 3.3 simulator. We also used Asertia TSA Crusher to analyze the evaluation time for obtaining a timestamp from a free TSA. Other WSN approaches for the monitoring of agriculture are described in [37,38,39].

## 4. The Trust WSN Model for Humidity and Moisture Monitoring

**Figure 1:**
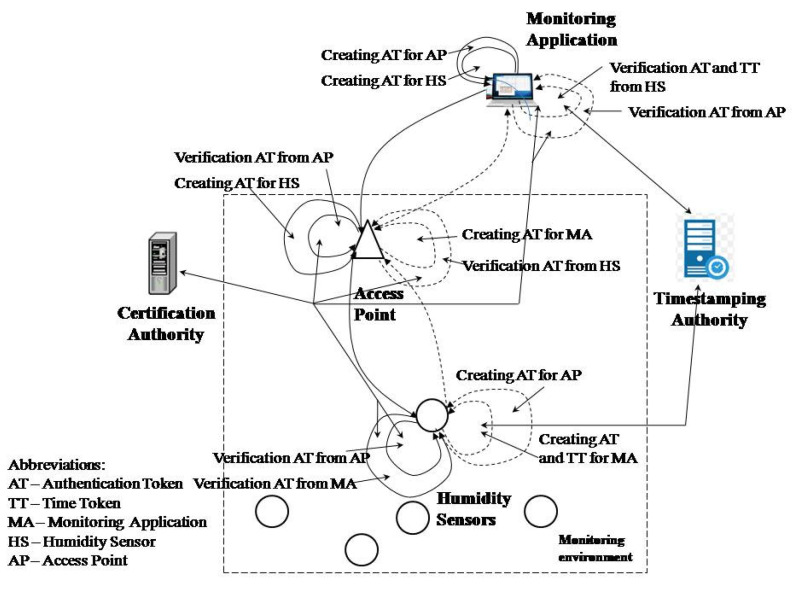
Model for trust in a WSN for humidity and moisture monitoring.

The PKI and TSA technologies were applied to establish trust between all WSN entities in the model. PKI technology was used to establish trust between entities in communication, while timestamp technology was used to build trust in time. The timestamp was not used by all entities in the network, but only by sensors in order to achieve trust that the sensor data had not been changed or created after the assigned time. The time dimension of trust was realized between the humidity sensor and the monitoring application. This allowed the monitoring application to have reliable information that the data were received from the original humidity sensor and were not generated or changed after the assigned time. Other entities participating in WSN communication were not assigned a time dimension of trust as the monitoring of the sending time has no impact on monitoring the environment. The model is shown in Figure 1.

Trust in the model was achieved through authentication and the time token and sender’s digital certificate. With the authentication token, the entities represent each other using a digital signature and their digital certificate. The recipient trusts the sender’s submitted data if the verification of the digital signature and the verification of the sender’s certificate is successful. The sender’s digital certificate verification involves checking the trust chain, the expiration time, and status of the certificate (entity, or TSA, and all participants in the trust chain). The time token was used to establish the time dimension of an event (the time of data creation in this model).

The monitoring application generates a trust request to read the sensor parameters. The trusted request contains an authentication token by which the monitoring application presents itself to the humidity or moisture sensor. The sensor checks whether there is trust in the monitoring application by verifying the digital signature based on the authentication token (a digital signature structure and monitoring application certificate). If the verification is performed successfully, then the sensor trusts the application and can be sure that the request was received from the monitoring application it trusts.

After creating an authentication token, the monitoring application for the sensor creates an authentication token for the access point (AP) to achieve trust with the AP during the transfer of data. The AP verifies the received authentication token. If verification is unsuccessful, the AP interrupts the transmission of messages to the sensor because it believes that the sending origin is suspicious. If the verification is successful, then the AP creates its authentication token which, along with the received message, is forwarded to the sensor.

The sensor verifies the authentication access point token. After successful verification, it verifies the authentication token of the monitoring application. The sensor establishes trust with the application if the verification of the authentication token of the monitoring application is successful. The sensor reads the humidity and prepares the authentication token and the timestamp token. The sensor sends a request to the TSA for a timestamp. The TSA prepares a trusted timestamp and sends it to the sensor. The sensor forms a trust structure and sends it to the monitoring application, along with the data. After creating a token for the application, the sensor creates an authentication token for the AP through which it sends data to the application. The AP trusts the sensor if it successfully verifies the authentication token received from the sensor. The AP then prepares the authentication token for the application and forwards the sensor message.

When the monitoring application receives a message from the AP, it performs verification of the AP token to determine if it has received a message from a trusted sender. The application rejects the message if the verification fails. If the AP token verification is successful, then the application performs sensor trust verification process, where it first verifies the authentication token and then verifies the trusted timestamp. If the verifications are successful, the monitoring application can be sure that the data were obtained from a sensor it trusts and that the data were not created or changed after the assigned time. The data reading time can be determined based on the timestamp and the established time required to obtain the timestamp from the TSA. The monitoring application does not establish trust with the sensor if the authentication token verification fails and the time token verification is successful. The data are rejected in this case, even if the time is successfully verified, because the sensor source cannot be stated with certainty. The monitoring application has confidence in the sensor and the obtained data if the authentication token is successfully verified and the time token is unsuccessful but has no trust at the time of occurrence for the data.

### 4.1. Prerequisites for Establishing a Trusted WSN

It is necessary to prepare (initialize) all network entities to establish trust in the WSN according to the model. The requirements for the PKI and TSA need to be defined before installing the necessary parameters in the confidential WSN entities. The requirements determine the choice of PKI and TSA that will be able to ensure the transfer of authority trust to the WSN entities, and thus the establishment of overall entity trust and trust at the time the data are generated.

#### 4.1.1. The Requirements for PKI and TSA Selection for the Establishment of a Trusted WSN

The basic requirements for the selection of the PKI and TSA are detailed below.

Selection of the PKI architecture: Choosing an adequate PKI architecture is a challenge. Each PKI architecture has advantages and disadvantages that should be taken into consideration [40]. PKI architectures of third parties (providers) provide services to a wide range of users. The selection of such a provider carries the risk of complex certificate management or the existence a large list of revoked certificates that would slow down the process of checking the certificate status. Building one’s own PKI architecture, on the other hand, is a complex process that requires consideration of a set of requirements from different areas [41].

The scheme of issuing a timestamp: Timestamp issuance schemes are classified into three types [42]: simple, chained, and distributed. Simple schemes are easy to implement and consist of a single TSA that issues timestamps. This scheme is the most appropriate choice to confirm time. Chained and distributed schemes are complicated because they generate complex timestamps which result in complex verification, as such a process requires direct involvement with the TSA.

The time required to process the request: The TSA server must send a response to the request within 1 min. Response time is the difference between the time the TSA receives the request and the time that is imprinted in the timestamp.

Compatibility with the PKI: When selecting a TSA, compatibility with the PKI should be considered regarding hash functions, cryptographic algorithms, and cryptographic keys. This is necessary due to the requirement of the implementation of a common system.

Key generation: Humidity sensors are devices with limited resources and do not have sufficient hardware resources to generate a random high-entropy private key. Consequently, it is desirable that the key pair and public and private keys be generated by the certification authority. The private key is installed on the entity, while the public key is contained in the entity’s digital certificate. It is necessary to consider whether to remove the entity’s private key from the certification authority database or to be safely stored in case of renewal.

Selection of the cryptographic algorithm: Cryptographic algorithms lead to high power consumption for sensors. As such, it is necessary to choose an appropriate algorithm to allow maximum operating autonomy for a sensor. In general, RSA algorithms demand more energy than ECC algorithms. ECC algorithms are suitable for sensors due to their higher energy efficiency and ability to provide the same level of security with a shorter key length; however, the choice of algorithm depends not only on its energy efficiency, but also on the power of the hardware and sensor’s power supply, as well as the given PKI for the sensor system.

Scalable database: A large number of sensors require an efficient and scalable system to manage databases that store certificates and other sensor data necessary for the functioning of the PKI. A PKI is a complex and expensive solution, so it can be expected to be used for different purposes and with a large number of end entities.

The digital certificate lifetime: The certificate lifetime may vary depending on the purpose of the certificate and the scenario in which it is used. Some authentication implementation scenarios require short-term certificates. To support this requirement, it is necessary to enable the issuance of certificates with different time, recall, and/or renewal deadlines.

The certificate management: The software of the certification and registration authority must be highly accessible, i.e., tolerant to faults [43] and scalable to ensure efficient management of certificates (specifically issuance, revocation, suspension). 

The certification path: Chains of certificates are achieved through trust relationships between certification authorities to determine whether the certificate being checked is signed by its publisher. A trust relationship is a link between the user’s certificate and the CA to which the user trusts, assuming that the CA has issued the appropriate valid certificate [44]. The long certification path is complex to validate and requires many resources. Certification paths with bidirectional trust relationships further complicate the problem of trust chain verification.

The certificate status management: The PKI architecture must allow the user to easily manage the status of the certificate without restriction via the registration authority. In this way, the user can effectively manage the trust of an entity.

The certificate structure planning: It is necessary to plan the content of the certificate, the name of the certificate owner, the certificate purpose and validity, the choice of the cryptographic algorithm, the length of the key, and the certificate status before applying for a certificate.

Checking certificate status: Checking the status of a certificate in the PKI can be performed with a certificate revocation list (CRL) and online certificate status protocol (OCSP). The CRL is a list generated by the certification authority at a certain time with a known validity period. The list consists of serial numbers of revoked and suspended certificates and times and dates of revocation, as well as the reasons for revocation. The CRL also contains some other information, such as the version, signature algorithm, issuer name, issue date of the CRL, and next update date. The CRL provides certificate management at the level of the validity period. The OCSP allows the statuses of certificates to be checked in real time. The advantage of the OCSP is real-time WSN trust management; however, such a check would slow down the response of the sensor and cause additional traffic in the network as the check would be performed by each WSN entity to verify the trust.

#### 4.1.2. The Entity Initialization

Each entity is issued a digital certificate with an appropriate validity period. The digital certificate and the corresponding private key are installed in the relevant entities. A digital certificate confirms the connection between the public key and the entity that owns it. Each entity needs to have a certificate chain installed to ensure trust in the certificate. The certificate chain is a series of digitally signed certificates issued by certification authorities. Certificate chain verification, i.e., verification of trust in authority, is necessary to determine whether a certificate (public key) that is used to perform authentication comes from the trusted authority. The trust chain verification activity is performed when verifying the authenticity of the entity that sent the message. There is no trust in the authority if only one link (certificate) does not pass the check and, thus, there is no trust in the entity that has the certificate.

### 4.2. Generating Tokens for Establishing Trust

An authentication token can be generated for all WSN entities for humidity and moisture monitoring. This token confirms the trust between the participants in the exchanged messages. The timestamp token establishes trust at the moment the data are created. The generation of a timestamp token is essential for the data source (sensor) and the monitoring application, as well as for the subsequent analysis of data.

**Figure 2:**
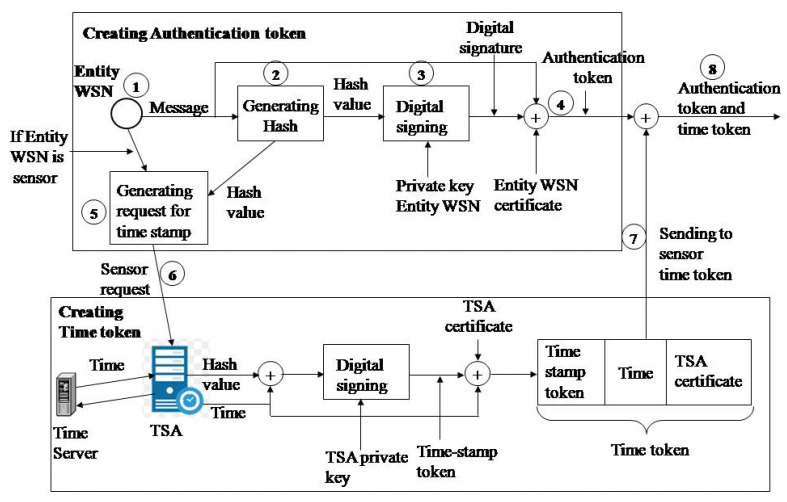
Generating the authentication token and time token.

An authentication token is generated when an entity presents itself to another entity. The algorithm used for generating the authentication token is shown in Figure 2 and is implemented in the following steps:

- Step 1. The original message is generated (sampling value of the sensor or the message sent by the access point (AP) or the message sent by the monitoring application, depending on which entity is being observed);
- Step 2. Calculating the hash value of the original message using the hash function (e.g., SHA-1);
- Step 3. The hash value of the original message is encrypted with the RSA algorithm and the private key of the entity (e.g., 1024 bits). A digital signature has now been created;
- Step 4. A new message format (authentication token) is created: original message, digital signature, entity certificate;

The algorithm for generating the timestamp token is shown in Figure 2 and is implemented through the following steps:

- Step 5. The sensor generates a timestamp request with the hash value obtained during the formation of the authentication token;
- Step 6. The sensor sends a TSA timestamp request;
- Step 7. Receives a response from the TSA (time token) in the following format: digitally signed timestamp token, time generated by the TSA, and digital TSA certificate;
- Step 8. Creating the authentication token and time token in the following data format: data, digital signature, sensor certificate, digitally signed timestamp token, time generated by TSA, and digital TSA certificate.

### 4.3. The Token Verification for Establishing Trust

The receiving entity verifies the confidentiality token. Authentication token verification is performed for all entities when receiving a message, while time token verification is only performed on the end entity to whom the message from the sensor is intended for, which is the monitoring application.

**Figure 3:**
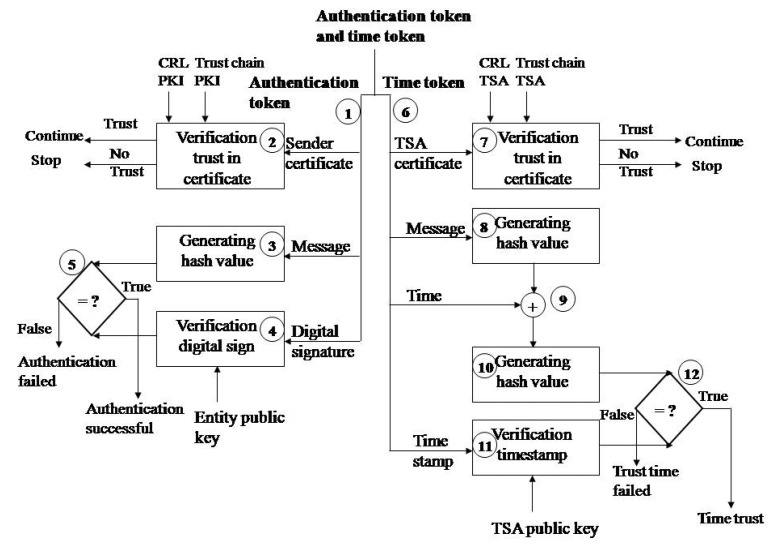
Verification of the authentication token and time token.

The authentication token verification on the side of the entity receiving the message is shown in Figure 3. The verification is performed as follows:

- Step 1. Deconcatenation of the authentication token to the original message, digital signature, and sender entity certificate;
- Step 2. Checking the validity of the sender’s certificate;
- Step 3. The same hash function (e.g., SHA-1) calculates the hash value of the original message;
- Step 4. The digital signature is decrypted by the RSA algorithm and the sender’s public key (from a digital certificate, e.g., 1024 bits);
- Step 5. The obtained values are compared. The digital signature is valid if the values are the same. This means that the message was sent by the entity currently being represented.

Timestamp verification is performed on the monitoring application side. Verification is performed through the following steps:

- Step 6. Deconcatenation of the time token to the TSA certificate, original message, time and timestamp;
- Step 7. Validation of the TSA certificate as described in below. The certificate chain is the TSA certificate and then the root CA certificate. The root CA signs the TSA certificate.
- Step 8. The same hash function (e.g., SHA-1) calculates the hash value of the source data (done during digital signature verification);
- Step 9. Concatenation of the calculated hash value and time;
- Step 10. The hash function calculates a hash value from the data hash value and time;
- Step 11. The decryption of a trusted timestamp with a public key from a TSA digital certificate;
- Step 12. Comparison of the obtained hash value and decryption content.

Checking the validity of the sender’s certificate represents checking the trust (i.e., whether there is trust in the issuer of the certificate), checking the status of the certificate, and checking the validity of the certificate:

- Verification of trust in the certificate authority: The certificate chain starts from a root CA certificate (highest trust point) via an intermediate CA certificate (this certificate is digitally signed with a CA root private key) and an entity certificate (the certificate is signed with an Intermediate CA private key). Verification is performed by verifying the digital signature of the root CA certificate and the Intermediate CA certificate using the CA root public key, while verification of the entity certificate digital signature is performed by using the Intermediate CA public key. If this can be done successfully, then there is trust in the certificate chain, i.e., that the certificate was issued to the entity by a trustworthy CA.
- The certificate status is checked based on whether the certificate has been revoked or suspended. Checking is performed for all certificates in the certificate chain. This is done by searching the certification revocation list (assuming that the lists (root CA CRL and intermediate CA CRL) are located at the entity).
- Certificate validation involves verifying the expiration date for all certificates in the certificate trust chain.

Validation of the TSA certificate is performed as described above. The certificate chain is now the TSA certificate and the root CA certificate. The root CA signs the TSA certificate.

## 5. Proposed Model Evaluation

The main goal of the simulation results described here is to evaluate the proposed model in terms of energy consumption at both receiver and sender nodes and in terms of packet delay when using SHA1 with the RSA-1024, RSA-2048, and RSA-3072 systems. The RSA cryptosystem uses a key of at least 1024 bits and is widely used due to the following reasons: First, RSA systems allow fast digital signature generation, which is suitable for applications where a network requires authentication for data. Second, they are compatible with existing communication infrastructure drives to adopt RSA systems in sensor networks [45]. The performance evaluation has been carried out in the Castalia 3.3 [46] simulator using a simple scenario consisting of one receiver and five sender nodes. Each sender node generated a data packet of 32 bytes at a load of 1 packet/s which was forwarded to the receiver, along with a digital signature and certificate using the MPQ-MAC protocol [47].

In clustering, a large network is sized into different clusters that are smaller in size with one receiver per cluster. This has many advantages, including the promotion of scalability and energy efficiency [48]. The performance of the proposed model was evaluated using a cluster consisting of one receiver and five sender nodes, which was sufficient to evaluate the characteristics of the proposed model; however, it could be extended to other clusters in the network. 

Table 1 summarizes the key parameters, including those of CC2420 radio and TelosB, which are widely used in sensor networks. 

### 5.1. Average Energy Consumption

**Figure 4:**
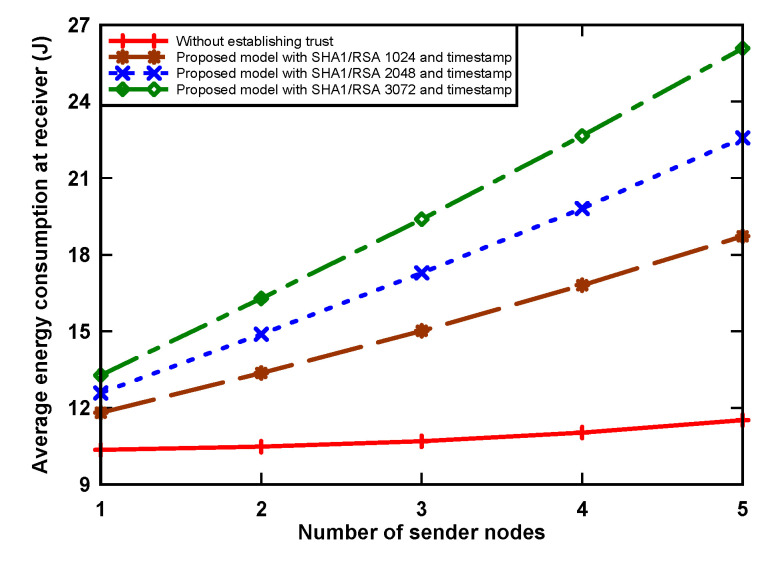
Average energy consumption of receiver node.

**Figure 5:**
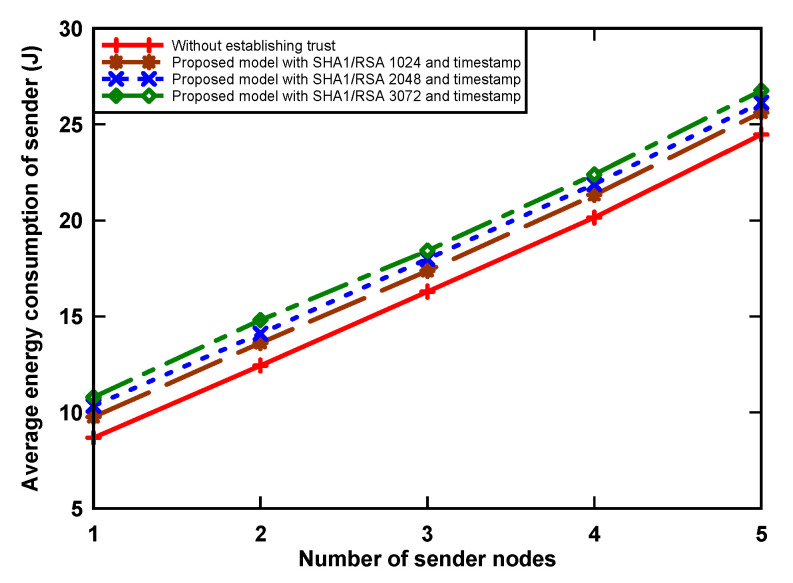
Average energy consumption of sender nodes.

Figure 4 and Figure 5 show energy consumption at the receiver and sender nodes, respectively, as incurred by digital signature algorithm that was implemented. The receiver node wakes up periodically and receives packets from contending nodes. The graph shows that the amount of energy consumption increased when the number of sender nodes increased. The reason for this is that the receiver receives more data when the sending number increases. We can also see that the proposed model uses more energy, i.e., up to 126.3% with RSA-3072 when compared to the non-signature data transmission scenario. This is because of two reasons: First, the receiver node receives a large number of packets from sender nodes, which incurs a significant amount of energy. Secondly, the sender nodes, having data packets, use a hash algorithm, asymmetric cryptographic algorithm, RSA-1024, RSA-2048, and RSA-3072 to provide authentication of the trust token by generating a data hash value and then signing using the private key from the sensors, which also increases the receiving time for data at the receiver side, and, as a result, an increase in consumption. Simultaneously for the sender node, when holding a data packet, the node incurs an energy use increase of up to 9.3% in the transmission of the original data, digital signature, and digital certificate. This is because the proposed model aims to provide trust, which incurs a slight increase in energy consumption due to the inherent overhead. 

The equation to calculate the energy consumption (*E_T_*) of node is given as follows:

(1) $$E_{T}\;=\;\sum_{i\;=\;0}^{n}P_{i\;}\times \;t_{i}$$

where *n* denotes the number of states, *i* is the CC2420 radio state, *P_i_* is the power consumption rate of state *i*, and *t_i_* is the time spent in state *i*.

### 5.2. Average Packet Delay 

**Figure 6:**
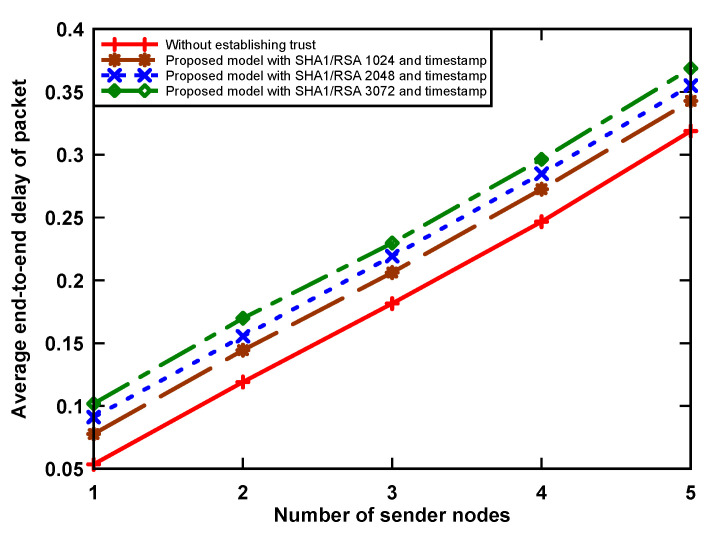
Average packet delay.

The end-to-end delay of a data packet is the sum of the queuing, transmission, propagation, and processing delays. Figure 6 shows the average delay of a data packet with and without the implementation of data security algorithms in the network. It can be seen that the implementation of an authentication trust token using the RSA-1024, RSA-2048, and RSA-3072 security algorithms on a sensor node increases packet delay by up to 15.6% when compared to a non-guaranteed data scenario, which is still within acceptable limits. This reason for this is that the transmission of original data, digital signature, and digital certificate incurs a small increase in packet delay. It can be noticed that the packet delay increases when the number of sending nodes is higher. This is because a sender node that holds a data packet has to wait a longer time to access the medium and packet delay increases as a result. 

### 5.3. Free Timestamping Authority Evaluation

We evaluated the possibility of applying the timestamp to achieve sensor trust in the time dimension by simulating the issuance of free-of-charge TSA software Ascertia TSA Crusher timestamps [49]. This software was used to test the efficiency of a TSA server by obtaining the total time consumed when assigning a timestamp as a result. Testing was performed with seven randomly selected free TSAs that the testing application could access. The selected TSAs are shown in Table 2.

We simulated several different environments for monitoring humidity and moisture sensors. A situation where one monitoring application required data from one or more sensors was simulated. A second simulation was related to two different monitoring environments with two monitoring applications operating simultaneously but independently requesting data from the same number of sensors. A third simulation featured three different monitoring environments where three monitoring applications simultaneously requested data from the same number of sensors.

The simulations were performed to test the response speed of the TSA server, i.e., to determine the time difference between the assigned timestamp for the first and last request. With this approach, we established the most suitable TSAs for the purpose of gaining trust in the time dimension for monitoring different humidity and moisture environments.

Simulations were performed with all seven selected free TSAs by measuring the total time consumed in seconds (TTC) and calculating the percentage of requests processed in the first second (PRP). The time consumption to generate the request was the same for each sensor, while the time to obtain the timestamp from the TSA differed depending on the order of arrival of the request, the speed of the TSA, and the load of the TSA requirements by third entities.

#### 5.3.1. Simulation of TSA Efficiency in Monitoring Humidity and Moisture Environment with One Application

**Figure 7:**
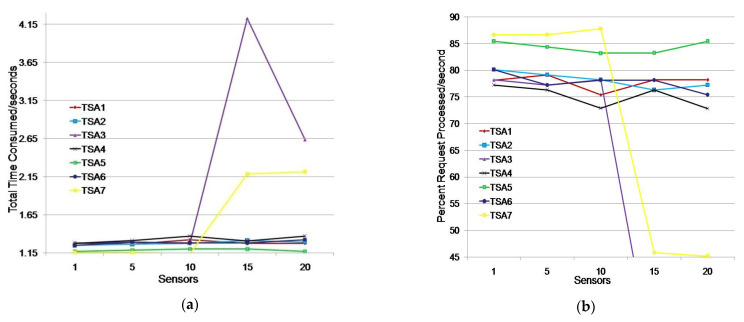
Issuing TSA timestamps for a single monitoring application environment: (**a**) total time consumed (s); (**b**) percentage of requests processed/s.

We considered a scenario where a monitoring application simultaneously searched for data from 1, 5, 10, 15, or 20 sensors. A sensor generated a request to obtain a timestamp when receiving a request from the monitoring application. The test results are shown in Table 3 and Figure 7.

The testing showed that all seven TSAs could generate timestamps for the listed sensor groups (Table 3). TSA5 had the fastest response for all tested sensor groups. The average TTC time was 1.186 s, i.e., timestamps were generated in the first second on average for 84.36% of the sensors (Figure 7). TSA7 showed better results than TSA5 for groups of up to 10 sensors, but it showed poor results for groups larger than 10 sensors. Its TTC increased by one second, which caused TSA7 to issue timestamps for only 45% of the sensors in the first second.

TSA1, TSA2, TSA4, and TSA6 showed similar results. They issued timestamps in 1.296 s and 77.23% of the sensors received a timestamp in the first second on average. TSA3 deviated significantly from other TSAs for requirements obtained from more than 10 sensors. 

We additionally tested TSA3 and TSA7 when 15 and 20 sensors sent a timestamp request. TSA3 did not respond within 60 s in 16% of cases. In 12% of cases, the results were similar to Table 3, while in 72% of cases the average TTC was 3.19% higher than the average TTC values of the observed TSAs in the scenario where 15 sensors requested a timestamp. TSA3 did not respond within 60 s in 24% of cases and it showed significantly worse results (4.223 s) in 24% of cases, and in 36% of cases it showed better results on average by 49.53% when compared to the results shown in Table 3 (2.636 s) in the scenario where 20 sensors requested a timestamp. The tests for TSA7 with 15 and 20 sensors requesting a timestamp showed that the average TTC values were 5.64% and 2.13%, respectively, which were higher than the average TTC values for TSA1, TSA2, TSA4, TSA5, and TSA6. Additional tests showed that TSA7 could be considered for assigning timestamps in a single monitoring application environment, while TSA3 with 15 and 20 sensor requests is not reliable due to non-response to requests. 

The test results indicate that TSA5 is the best choice for assigning timestamps to sensors in a single monitoring application environment because of its average timestamp issuance time being 8.49% higher than the average time of a continuous group (TSA1, TSA2, TSA4, and TSA6), while its average time for issuing timestamps was 20% higher than the average time of all other TSAs. TSA7 should be used to issue timestamps for environments of up to 10 sensors.

#### 5.3.2. Simulation of TSA Efficiency for Monitoring Humidity and Moisture Environment with Two Applications

**Figure 8:**
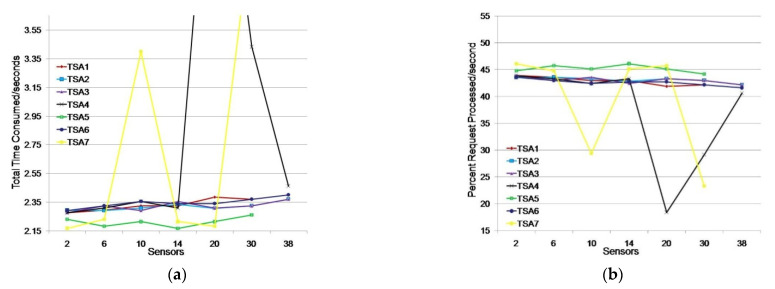
Issuing timestamps for TSAs for an environment with two monitoring applications: (**a**) total time consumed (s); (**b**) percentage of requests processed/s.

We considered a scenario where two monitoring applications simultaneously searched for data from 1, 3, 5, 7, 10, 15, or 19 sensors. We conducted testing with an additional TSA load by having two independent monitoring applications which request data from the same number of sensors (e.g., 15 sensors per application or a total of 30 sensors). The test results are shown in Table 4 and Figure 8.

The test results showed that three TSAs (TSA1, TSA5, and TSA7) did not respond to the simultaneous request of two groups of 19 sensors to issue a timestamp. Other TSAs generated requests with approximately the same response time. TSA7 and TSA4 showed oscillations at the time of timestamp issuing by 16.08% and 21.53%, respectively, compared to the average time of the other TSAs in the moment of testing. 

By additional TSA7 testing for the requirements of 10 and 30 sensors we obtained average TTC values of 2.247 and 2.495 s, respectively. The obtained average TTC value for 10 sensors is better than the average TTC value of the observed TSAs (2.309 s), while the obtained average TTC value for 30 sensors is slightly worse. By testing TSA4 for 20 sensors, the average TTC value obtained was 2319 s, slightly higher than the average TTC value of the observed sensors, 2.291 s. Testing TSA4 for 30 sensors showed next results: TSA4 did not respond to sensor requests in 36% of cases, while in 64% of cases it responded with a slightly worse average TTC value (2.426 s) compared to the average TTC value of TSA1, TSA2, TSA3, TSA5 and TSA6 (2.340 s). Additional testing indicates that the oscillations in TSA7 and TSA4 were instantaneous, however the problem is that TSA4 did not respond to the requirements of 30 sensors in 36% of cases.

TSA5 showed the best results with an average timestamp issuance time of 2.213 s, which was 4.57% better than TSA2, which was ranked second; however, TSA5 did not respond to the simultaneous request of monitoring applications (two groups of 19 sensors). Continuous stability in timestamp issuing was achieved by TSA2, TSA3, and TSA6, with an average timestamp issuance time of 2.329 s and an average timestamp issue in the first second for 42.94% of sensor requests.

#### 5.3.3. Simulation of TSA Efficiency for Humidity and Moisture Environment with Three Monitoring Applications

**Figure 9:**
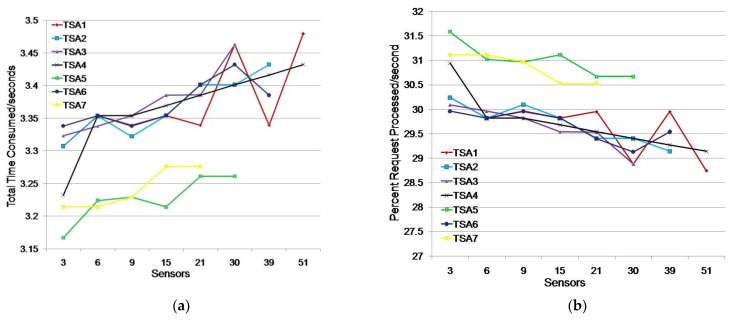
Issuing TSAs timestamps for an environment with three monitoring applications: (**a**) total time consumed (s); (**b**) percentage of requests processed/s.

We considered a scenario where each of the three monitoring applications simultaneously searched for data from 1, 2, 3, 5, 7, 10, 13, and 17 sensors. We further loaded the TSAs by having three independent monitoring applications simultaneously request data from sensors (e.g., three groups of 13 sensors each or a total of 39 sensors). The test results are shown in Table 5 and Figure 9.

The test results show that only two TSAs generated timestamps for the maximum number of sensors (51 sensors), while four TSAs generated timestamps for 39 sensors. All of the TSAs considered in this scenario generated timestamps for the requirements of 21 sensors.

Figure 9 shows three groups of TSAs along with times spent issuing timestamps. The first group consists of TSA5 and TSA7, which showed the best results with average timestamp issuance times of 3.226 s and 3.242 s, respectively, i.e., 31.00% and 30.85% of timestamps issued in the first second, respectively. The second group consists of TSA2, TSA3, TSA4, and TSA6, which oscillate around a mean value of 3.371 s of time spent on issuing timestamps, with 29.68% of timestamps issued in the first second. The third group includes TSA1, which achieved the largest oscillations in the time spent issuing timestamps; however, its average time for issuing timestamps was almost identical to the other group, so we should not dismiss it as unreliable.

The simulations of TSA efficiency for humidity and moisture environments showed that the upper limit of efficiency for obtaining a timestamp from a free TSA is 30 sensors. The reason for this pertains to the load on a free TSA by other users. It is necessary to group sensors into clusters of up to 30 sensors and then assign a different free TSA to each cluster in environments with large numbers of sensors. Another solution is to establish an internal TSA. This is a more expensive solution, although it would be more reliable and efficient solution for environments with large numbers of sensors.

### 5.4. Security Evaluation

The security evaluation of the proposed model was based on the following criteria: trust authenticity, trust integrity, trust non-repudiation, time dimension of trust, comprised sensor private key, compromised trusted service provider, long-term trust, certification authority and TSA selection, and disturbance in the WSN environment.

Trust authenticity: Trust authenticity was based on the authenticity of the parties in the model using a digital certificate and a digital signature. Party authentication violation in the proposed model was observed through the following cases: entity digital certificate content change, falsification of the entity digital certificate, changes in the trust chain, falsification of the trust chain, or changes of the digital signature.

- The attacker cannot jeopardize the established trust by changing the content of the entity digital certificate because checking the trust chain on the recipient side would determine that the certificate content of the sender sensor has changed. The transaction would be rejected as untrustworthy because it comes from an untrustworthy source.
- Falsification of a digital certificate means that there is a digital certificate with the same content but signed by a fake certification authority. It would be established in the process of the certificate chain verifying that the certificate was not issued by a certification authority that is trusted. As a consequence of the above, a sensor that has such a certificate would not be trusted.
- Changing or falsification of the trust chain cannot jeopardize trust because checking the trust chain would reveal that it has not been issued by a certification authority that the entity trusts.
- The digital signature changes cannot affect trust because the applied asymmetric cryptography in the digital signature verification process would indicate that there has been a change in the digital signature. This indicates that the authentication token or certificates in the trust chain are not signed by the party that has the private key corresponding to the public key in the certificate, or the digital signature has been maliciously altered to avoid establishing trust.

Trust integrity: Trust integrity is based on the authentication token integrity, i.e., the message, digital certificate, and digital signature. Changes in data, digital certificates, or digital signatures cannot jeopardize the trust model in terms of the trust imposition by the attacker. The model integrity is preserved through the verification of the trust chain and digital signature, as well as the application and features of the hash function used in digital signing.

Trust non-repudiation in the model: Trust non-repudiation in the model is based on the established chain of trust. If the trust chain to the trusted service provider is disrupted in any of the ways described above, the entrusted entities may now deny the established trust.

Time dimensions of trust: The time dimension of trust is based on the integrity of the time token and the TSA digital certificate. Attacks targeted at changing the content of the token or TSA digital certificate will jeopardize trust in the time dimension of the model. Such attacks are detected during the first verification of the time token or TSA digital certificate. The time dimension of trust, as well as the availability of the model, is affected by the violation of the timestamp request integrity such that any invalid TSA timestamp request is denied.

Compromised sensor private key: Compromising the sensor private key implies that the attacker has obtained the sensor private key in an unauthorized manner. The private key is used to digitally sign the message and create an authentication token. Having a sensor private key and a digital certificate allows an attacker to create a fake sensor that will generate fake data, and the entire system in which the sensor is located will trust to it. The work of [32] contains a proposal to improve sensor hardware in order to overcome this problem. In this case, it is necessary to immediately revoke the certificate of the compromised sensor and thus exclude it from the trust model.

Compromised trusted service provider: The model relies on two trusted service providers: a certification authority and a TSA. If an attacker compromises the certification authority’s private key or TSA’s private key, then the TSA is initiated with a new private key, all certificates issued by the certification authority are revoked, new certificates are issued to model participants, and a new trust is initiated.

Long-term trust: The model not only enables trust in the data source (sensor) and the time of data creation at the moment of processing immediately after receiving the data, but also enables trust after a long time. Such data stored in the database may cause the attacker to modify them. There would be unforeseeable consequences in the processes of agricultural and industrial production if a decision was made on the basis of changed data. The mechanism of the authentication token and the time token enables the detection of subsequent malicious changes.

Selection of the certification authority: It is necessary to choose a trustworthy certification authority that will provide secure certificates for WSN participants in order to establish the trust in the proposed model. The main weakness of a public PKI is that any certification authority can sign a certificate for any resource or sensor. Public certification authorities are located in many countries and some of them are potentially hostile. In such countries, certification authorities may be forced to issue certificates to non-guaranteed users. Such users can use fake certificates for the purposes of espionage, the falsification of messages, or false representation. It is necessary to use certificates issued by verified certification authorities that are accredited or internal certification authorities established for the model in order to avoid this situation. These certification authorities guarantee that no one else can issue certificates on their behalf, and thus reliability in the trust model.

TSA selection: The TSA selection has an indirect impact on trust in the model security. If a free TSA choice is made such that it is overloaded by other users or if it often does not meet the timestamp requirements, then this will affect the availability of the model. This problem is best overcome by testing free TSAs and only choosing free TSAs that have the best performance with a small number of failed timestamp issues, or otherwise an internal TSA should be established for one’s own needs.

Disturbance in WSN environment: The deployment of a WSN in outdoor applications, e.g., agriculture, may expose the WSN to a constantly changing environment, which can affect the data transmission in the network. For instance, changes in surrounding temperature disturb link quality and radio communication and can result in packet loss. Thus, it is suggested to protect sensor nodes from high temperatures by placing them in shaded environments in case nodes are fully operated through battery power. One may also consider the surrounding conditions while deploying nodes [50]. Disturbances in the environment can cause a change in the authentication data transmitted in a WSN. The model considered here effectively detects such changes on the receiving side. This means that the entity receiving the message knows that there has been a change in the data in the transmission with certainty, so it cannot trust the party that sent the data. If the sensor provides incorrect data due to poor environmental conditions, then the receiving entity will have trust in the sensor that sent the data. The analysis of the data determines whether they are relevant or not, but it is important to determine whether the data originate from a real sensor and not a fake one.

## 6. Conclusions

This research has evaluated the possibility of applying an authentication mechanism based on asymmetric cryptography, a PKI, and time dimension using free TSA timestamps for the realization of a trusted WSN for monitoring humidity and moisture environments. The proposed research has included the development and evaluation of a model and shows that the model is effective for establishing a trusted WSN.

Research has shown that the time dimension can be considered when establishing trust. By establishing this element of trust, the monitoring application can be sure that data received from a sensor are not changed after receiving a timestamp from the TSA, as well as certainty that the sensor has generated the data no later than the time detailed the timestamp. Likewise, research has shown that a digital signature and assigned digital certificate can be used to establish trust between entities in WSN communication.

The results of the research indicate several facts that should be taken into account when applying this model. First, trust is established using a timestamp and an authentication token based on a digital signature. The application of an asymmetric cryptographic algorithm with a longer key length (e.g., RSA-3072) causes an increase in power consumption during transmission of up to 9.3% and up to 126.3% when receiving. It also increases the delay in sending a packet by 15.6% when compared to a packet system without a trust mechanism.

Second, free TSAs have shown stability in issuing timestamps of up to 30 simultaneous sensor requests; however, scenarios with multiple applications for monitoring a humidity and moisture environment through a trusted WSN showed longer mean waiting times of up to 3.331 s to obtain a timestamp and a lower percentage of issued timestamps, namely, up to 30.03% in the first second. The application of the model with one application for monitoring the humidity and moisture environment through a trusted WSN showed significantly better results, where the average time to obtain a timestamp was up to 1.440 s, while 74.67% of timestamps were issued in the first second on average.

Third, it is necessary to test free TSAs for issuing timestamps for a different number of sensors when implementing this model. Research has shown that some free TSAs give significantly better timestamp issuance times by an average of 17.64% when compared to other TSAs.

This research has shown that the model is applicable and features acceptable energy losses for the transmitting and receiving sides, with less delay in data transmission and a satisfactory time for obtaining the timestamp from a free TSA. In addition to the benefits of entity trust in the WSN for monitoring humidity and moisture, the model also has benefits regarding data integrity checks, non-repudiation in message exchange, and the impossibility to subsequently change data and falsify the time of data generation.

## Figures and Tables

**Table 1:** Simulation parameters.

Parameter	Value
Simulation time	1000 s
Transmission power	57.42 mW
Receiving power	62.04 mW
Idle listening power	62.04 mW
Sleeping power	1.4 mW
Size of data packet	32 bytes
Packet rate	1 packet/s
Sender nodes	1 to 5
Listen interval	60 ms
Duty cycle	0.5
Bandwidth	250 kbps

**Table 2:** Free timestamping authorities.

Short TSA Name	TSA URL
TSA1	http://zeitstempel.dfn.de (accessed on 3 March 2021)
TSA2	http://timestamp.apple.com/ts01 (accessed on 3 March 2021)
TSA3	http://time.certum.pl (accessed on 3 March 2021)
TSA4	http://adobe-timestamp.geotrust.com/tsa (accessed on 3 March 2021)
TSA5	http://tsa.swisssign.net (accessed on 3 March 2021)
TSA6	http://timestamp.entrust.net/TSS/RFC3161sha2TS (accessed on 3 March 2021)
TSA7	http://rfc3161timestamp.globalsign.com/advanced (accessed on 3 March 2021)

**Table 3:** TTC and PRP results for timestamp issuing of TSAs in a single monitoring application environment.

Sensors	Measured Values	TSA1	TSA2	TSA3	TSA4	TSA5	TSA6	TSA7
1	TTC (s)	1.280	1.248	1.279	1.295	1.170	1.248	1.154
	PRP (%)	78.13	80.13	78.19	77.22	85.47	80.13	86.66
5	TTC (s)	1.264	1.264	1.295	1.311	1.185	1.295	1.154
	PRP (%)	79.11	79.11	77.22	76.28	84.39	77.22	86.66
10	TTC (s)	1.326	1.279	1.279	1.372	1.202	1.280	1.139
	PRP (%)	75.41	78.19	78.19	72.89	83.19	78.13	87.80
15	TTC (s)	1.279	1.311	1.315	1.310	1.201	1.280	1.301
	PRP (%)	78.19	76.28	76.05	76.34	83.26	78.13	76.86
20	TTC (s)	1.279	1.295	1.306	1.373	1.170	1.326	1.366
	PRP (%)	78.19	77.22	76.60	72.83	85.47	75.41	74.85
Average value	TTC (s)	1.286	1.279	1.295	1.332	1.186	1.286	1.217
	PRP (%)	77.81	78.19	77.24	75.11	84.36	77.80	82.56

**Table 4:** Results of TTC and PRP timestamp issuing of TSAs in an environment with two monitoring applications.

Sensors	Measured Values	TSA1	TSA2	TSA3	TSA4	TSA5	TSA6	TSA7
2	TTC (s)	2.277	2.294	2.277	2.278	2.231	2.293	2.169
	PRP (%)	43.92	43.59	43.92	43.90	44.82	43.61	46.10
6	TTC (s)	2.294	2.293	2.324	2.309	2.184	2.325	2.231
	PRP (%)	43.59	43.61	43.03	43.31	45.79	43.01	44.82
10	TTC (s)	2.325	2.309	2.293	2.356	2.215	2.356	3.401
	PRP (%)	43.01	43.31	43.61	42.44	45.15	42.44	29.40
14	TTC (s)	2.324	2.335	2.355	2.309	2.168	2.340	2.215
	PRP (%)	43.03	42.83	42.46	43.31	46.13	42.74	45.15
20	TTC (s)	2.387	2.308	2.309	5.429	2.215	2.340	2.184
	PRP (%)	41.89	43.33	43.31	18.42	45.15	42.74	45.79
30	TTC (s)	2.371	2.324	2.324	3.432	2.262	2.371	4.290
	PRP (%)	42.18	43.03	43.03	29.14	44.21	42.18	23.31
38	TTC (s)	-	2.371	2.372	2.462	-	2.402	-
	PRP (%)	-	42.18	42.16	40.62	-	41.63	-
Average value	TTC (s)	2.330	2.319	2.322	2.939	2.213	2.347	2.748
	PRP (%)	42.94	43.12	43.07	37.31	45.21	42.62	39.10

**Table 5:** Results of TTC and PRP timestamp issuing of TSAs in an environment with three monitoring applications.

Sensors	Measured Values	TSA1	TSA2	TSA3	TSA4	TSA5	TSA6	TSA7
2	TTC (s)	3.307	3.307	3.323	3.232	3.167	3.338	3.214
	PRP (%)	30.24	30.24	30.09	30.94	31.58	29.96	31.11
6	TTC (s)	3.354	3.353	3.338	3.354	3.224	3.354	3.214
	PRP (%)	29.82	29.82	29.96	29.82	31.02	29.82	31.11
10	TTC (s)	3.339	3.322	3.354	3.354	3.229	3.338	3.229
	PRP (%)	29.95	30.10	29.82	29.82	30.97	29.96	30.97
14	TTC (s)	3.354	3.354	3.385	3.369	3.214	3.354	3.276
	PRP (%)	29.82	29.82	29.54	29.68	31.11	29.82	30.53
20	TTC (s)	3.339	3.401	3.386	3.385	3.261	3.401	3.276
	PRP (%)	29.95	29.40	29.53	29.54	30.67	29.40	30.53
30	TTC (s)	3.463	3.401	3.463	3.401	3.261	3.432	-
	PRP (%)	28,88	29.40	28.88	29.40	30.67	29.13	-
39	TTC (s)	3.339	3.432	-	3.416	-	3.385	-
	PRP (%)	29.95	29.14	-	29.27	-	29.54	-
51	TTC (s)	3.479	-	-	3.432	-	-	-
	PRP (%)	28.74	-	-	29.14	-	-	-
Average value	TTC (s)	3372	3.367	3.375	3.368	3.226	3.372	3.242
	PRP (%)	29,67	29.70	29.64	29.70	31.00	29.66	30.85

## Data Availability

Data is contained within the article.
